# Pathological examination of factors involved in PD-L1 expression in patients with oral tongue squamous cell carcinoma

**DOI:** 10.1186/s40902-024-00441-w

**Published:** 2024-08-08

**Authors:** Yu Koyama, Chiharu Ogawa, Chihiro Kurihara, Nao Hashimoto, Shota Shinagawa, Hiroya Okazaki, Takumi Koyama, Keisuke Sugahara, Akira Katakura

**Affiliations:** 1 Department of Oral Pathobiological Science and Surgery, Tokyo Dental College, Tokyo, Japan, 2-9-18 Kanda Misaki-Cho, Chiyoda-Ku, Tokyo, Japan; 2 Department of Oral and Maxillofacial Surgery, Okinawa Prefectural Chubu Hospital, Okinawa, Japan, 281 Miyazato, Uruma-Shi, Okinawa, Japan

**Keywords:** PD-L1, CAFs, α-SMA, p53, Oral squamous cell carcinoma

## Abstract

**Background:**

Tumor tissues comprise cancer cells and stromal cells, and their interactions form the cancer microenvironment. Therefore, treatments targeting cells other than cancer cells are also actively being developed, and among them, treatment targeting PD-1, an immune checkpoint molecule that is important in tumor immune evasion, has also been indicated for head and neck cancer. PD-L1, a ligand of PD-1, is expressed in both tumor cells and stromal cells, and the scoring system based on the combined positivity rates of both types of cells, the combined positive score (CPS), is used for predicting treatment effect. However, much is unknown regarding the expression of PD-L1. In this study, we histopathologically examined factors controlling the expression of PD-1/PD-L1. This study included 37 patients who underwent resection surgery for tongue squamous cell carcinoma in the Department of Oral and Maxillofacial Surgery at Tokyo Dental College Suidobashi Hospital. The expression levels of PD-L1, α-SMA, and p53 were assessed by immunohistochemical staining.

**Results:**

Seven participants had CPS ≥ 20, twenty-four participants had 1 ≤ CPS < 20, and six participants had CPS < 1. The overall positivity rate of α-SMA, a marker for cancer-associated fibroblasts (CAFs), was 27% (10/37 participants), and the positivity rates of α-SMA for the three CPS groups were 85.7% (6/7 participants), 16.7% (4/24 participants), and 0% (0/6 participants), respectively. In addition, the overall positivity rate of p53 was 37.8% (14/37 participants), and the positivity rates of p53 for the three CPS groups were 71.4% (5/7 participants), 37.5% (9/24 participants), and 0% (0/6 participants), respectively.

**Conclusions:**

The expression of PD-L1 demonstrated an association with α-SMA and p53 positivity. In addition, compared with the expression of p53, the expression of α-SMA demonstrated a higher association with PD-L1 expression in patients with a high CPS. The abovementioned findings suggest that the interactions between CAFs, cancer cells, and immunocompetent cells may regulate the expression of PD-L1.

## Background

Tumor tissue is comprised of cancer cells and stromal cells, including fibroblasts, vascular endothelial cells, and immune cells, shaping the cancer microenvironment. Numerous studies have reported the influence of stromal cells on tumor progression [1]. Tumor cells also activate stromal cells [2, 3]; and, a large body of evidence exist indicating that cancer cells and stromal cells interact forming the cancer microenvironment. Recent therapeutic approaches, particularly targeting T cells, such as immune checkpoint inhibition and chimeric antigen receptor (CAR)-T therapy, have emerged.

Programmed cell death protein 1 and programmed cell death ligand 1 (PD-1/PD-L1) inhibitors have been developed as postoperative chemotherapy for various cancer types, and numerous clinical trials have shown that they have high therapeutic efficacy [4]. PD-1 is expressed on cytotoxic T cells, and PD-L1 is widely expressed on cancer cells and stromal cells [5]. PD-L1 overexpression in cancer cells is considered to bind with PD-1 and induce tumor immune evasion via T cell exhaustion. For cases of oral cancer, PD-L1 overexpression in cancer cells correlates with cervical lymph node metastasis and poor prognosis [6, 7]. Immune checkpoint therapy targeting PD-1 is indicated in head and neck cancer, with PD-L1 expression often utilized for predicting treatment with immune checkpoint therapy [6]. Tumor proportion score, which is calculated from tumor cell positivity, and combined positive score (CPS), which evaluates both tumor and stromal cells, have been utilized for lung cancer [7]. For head and neck cancer, CPS has been employed in clinical trials in checkmate-048. Immunotherapy has been reported to offer a significantly better prognosis than conventional molecular targeted drugs in patients with CPS of 20 or higher, suggesting the clinical utility of CPS as a predictor of treatment response [8].

However, CPS alone has not been established as a marker, and the expression mechanism of PD-L1 remains unclear. We hypothesized that the interaction between cancer and stromal cells affects PD-L1 expression. In this study, we aimed to determine regulatory factors involved in PD-L1 expression in patients with oral tongue squamous cell carcinoma using immunohistochemical staining.

## Methods

### Subjects

Thirty-seven patients who underwent resection of oral squamous cell carcinoma of the tongue at Tokyo Dental University Suidobashi Hospital between 2016 and 2020 were included.

#### Immunostaining

PD-L1 staining was conducted via PD-L1 immunohistochemistry (IHC) 28–8 pharmDx (SK00521-5 J, Agilent Technologies, Santa Clara, CA). Tissues fixed in 10% neutral buffered formalin were paraffin-embedded, and tissue specimens were subsequently thinly sliced to 4–5 μm and placed on coated slides. Tissue sections were deparaffinized, dehydrated, and antigen-activated via the PT Link pretreatment system (Agilent Technologies, Santa Clara, CA). The sections were then placed in an automated staining machine, Autostainer Link 48 (Agilent Technologies, Santa Clara, CA), and stained automatically using the PD-L1 IHC 28–8 pharmDx protocol. Evaluation was conducted by a pathologist and scored using the CPS, which was calculated as follows.

CPS = PD-L1 positive cells(tumor cells, lymphocytes, macrophage)/viable tumor cells × 100 Since positive cells were evaluated in epithelial and stromal cells in Carcinoma in Situ (CIS) cases, CPS was calculated in the same way as for invasive carcinoma.

IHC was performed using anti-p53 antibody (DO-7, Agilent Technologies, Santa Clara, CA), and anti-α-smooth muscle actin (SMA) antibody (1A4, Agilent Technologies, Santa Clara, CA) as previously described [9]. Semiquantification of p53-positive tumor cell and α-SMA-positive fibroblasts was performed using the following steps. Five different fields in base membrane (p53) and fibroblast-rich stroma (α-SMA) of cancerous region were captured on each slide under 400 × magnification with a microscope. P53 expression was considered positive when ≧10% of the tumor cells nuclei by reference to previously described [10]. α-SMA expression was considered 10% of fibroblast-rich stroma area. Fibroblast-like cells were morphologically distinguished from both tumor and stromal cells, such as leukocytes and vascular endothelial cells. Since α-SMA positive fibroblast-like cells and p53-positive cells were also identified in CIS and early invasive carcinoma, they were included in the study.

### Statistical methods

Statistical analysis utilized the × 2 test, with level of statistical significance set to *p* < 0.05. We divided the T classification into T1 or T2 and the N classification into positive or negative for statistical analysis. The depth of invasion (DOI) was classified based on 4 mm, and the clinical inspection was classified endophytic or extrovert, taking into account a risk factor for cervical lymph node metastasis. And We All data were processed using the IBM SPSS software package ver. 23. (Chicago, IL, USA).

## Results

Among the 37 patients included, there were 18 males and 19 females, with a median age of 50 years; patient information is summarized in Table 1. Patients were categorized into three CPS groups and compared based on clinical characteristics (Table 2). Comparison of the two groups using patient gender and median age as cutoffs revealed no significant differences in PD-L1 expression. In T classification, 26 and 11 patients were T1 and T2 cases, respectively, indicating a significant difference between the three groups (*p* < 0.01). A significant difference was also found between the two groups using a DOI of 4 mm as the cutoff for prophylactic neck dissection according to National Comprehensive Cancer Network guidelines (*p* < 0.02).

**Table 1:** Clinical information of the 37 patients

Patient No	Sex	Age	T classification	N classification	DOI	Clinical inspection	CPS	p53	α-SMA
1	M	45	T2	N0	8	endophytic	47.5	+	+
2	F	63	T2	N0	2.8	extrovert	75	+	+
3	F	42	T2	N0	9	endophytic	65	+	+
4	M	84	T2	N0	2.2	extrovert	42.5	ー	+
5	F	41	T1	N0	1	endophytic	23	+	ー
6	F	50	T2	N0	5	extrovert	115	+	+
7	M	30	T2	N0	9	extrovert	23	ー	+
8	F	54	T1	N0	0.5	extrovert	12.5	ー	ー
9	M	60	T2	N0	CIS	extrovert	13	ー	ー
10	F	49	T1	N0	0.5	extrovert	11	ー	ー
11	M	71	T1	N0	1.5	extrovert	8	+	+
12	F	63	T1	N0	3.5	endophytic	6	ー	ー
13	F	47	T1	N0	CIS	extrovert	7	ー	+
14	M	52	T2	N2b	7.3	endophytic	6	+	+
15	F	79	T1	N0	4	extrovert	6	+	ー
16	M	62	T1	N0	0.1	extrovert	8.5	ー	+
17	F	25	T1	N0	CIS	endophytic	4	ー	ー
18	M	56	T1	N0	CIS	extrovert	3	ー	ー
19	M	61	T1	N0	1.5	extrovert	1	+	ー
20	F	42	T1	N0	CIS	extrovert	1	ー	ー
21	F	63	T1	N0	4	extrovert	1	+	ー
22	F	36	T1	N0	1	extrovert	3	ー	ー
23	M	54	T1	N0	CIS	extrovert	7	ー	ー
24	M	39	T1	N0	1.5	extrovert	2	+	ー
25	M	55	T1	N0	2.3	extrovert	12	ー	ー
26	F	65	T1	N0	0.5	extrovert	5	+	ー
27	M	27	T2	N0	1.2	extrovert	3	+	ー
28	F	41	T1	N0	0.5	extrovert	3	ー	ー
29	F	76	T2	N0	0.5	endophytic	1	ー	ー
30	F	64	T2	N0	0.5	extrovert	13	ー	ー
31	F	65	T1	N0	0.5	extrovert	15	ー	ー
32	M	43	T1	N0	0.8	extrovert	0	ー	ー
33	M	30	T1	N0	CIS	extrovert	0	ー	ー
34	M	45	T1	N0	CIS	extrovert	0	ー	ー
35	M	60	T1	N0	CIS	extrovert	0	ー	ー
36	F	63	T1	N0	CIS	extrovert	0	ー	ー
37	M	78	T1	N0	CIS	extrovert	0	ー	ー

**Table 2:** Comparison of combined positive score groups for each patient's clinical information

Variables		All Patients, No	Patients, No			*P* Value
			CPS < 1	1≦CPS < 20	CPS≧20	
Sex	Male	18	5	10	3	0.17
	Female	19	1	14	4	
Age	50≦	22	3	16	3	0.46
	< 50	15	3	8	4	
T classification	T1	26	6	19	1	< 0.01
	T2	11	0	5	6	
N classification	N(-)	36	6	23	7	0.75
	N( +)	1	0	1	0	
DOI	4 mm≦	7	0	3	4	< 0.017
	< 4 mm	30	6	19	3	
Clinical inspection	Endophytic	7	0	4	3	0.12
	Extrovert	30	6	20	4	

**Fig. 1:**
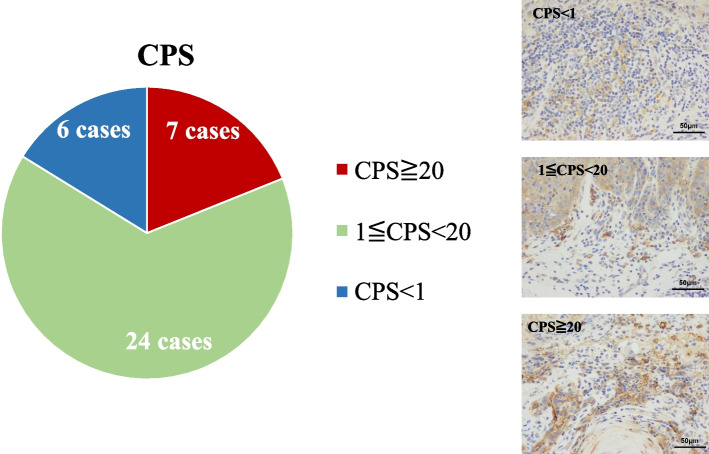
Number of patients in each CPS group evaluated via immunohistochemistry. Upper: Representative case of CPS < 1, magnificationX400, scale bar 50 μm. Middle: Representative case of 1≦CPS < 20, magnificationX400, scale bar 50 μm. Bottom: Representative case of 20≦CPS, magnificationX400, scale bar 50 μm

PD-L1 staining results showed the following: CPS ≥ 20 in 7 patients (18.9%), 1 ≤ CPS < 20 in 24 patients (64.9%), and CPS < 1 in 6 patients (16.2%) (Fig. 1).

**Fig. 2:**
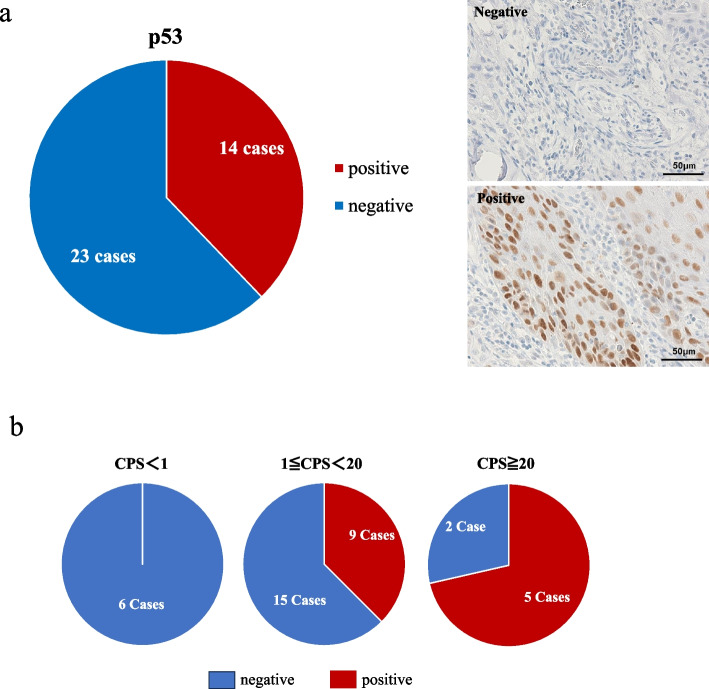
**a** Mutant p53 positivity in immunohistochemistry. Upper: Representative case of p53 negative, magnificationX400, scale bar 50 μm. Lower: Representative case of p53 positive, magnificationX400, scale bar 50 μm. **b** Percentage of mutant p53 positive in each CPS group

Immunohistological evaluation p53 mutations, the most well-known tumor suppressor gene, revealed mutations in 14 cases (37.8%) (Fig. 2a). The mutant p53 positivity rates for each CPS value were 71.4% (5/7) for CPS ≥ 20, 37.5% (8/24) for 1 ≤ CPS < 20, and 0% (0/6) for CPS < 1, revealing a significant difference (*p* < 0.03) among the three groups (Fig. 2b).

**Fig. 3:**
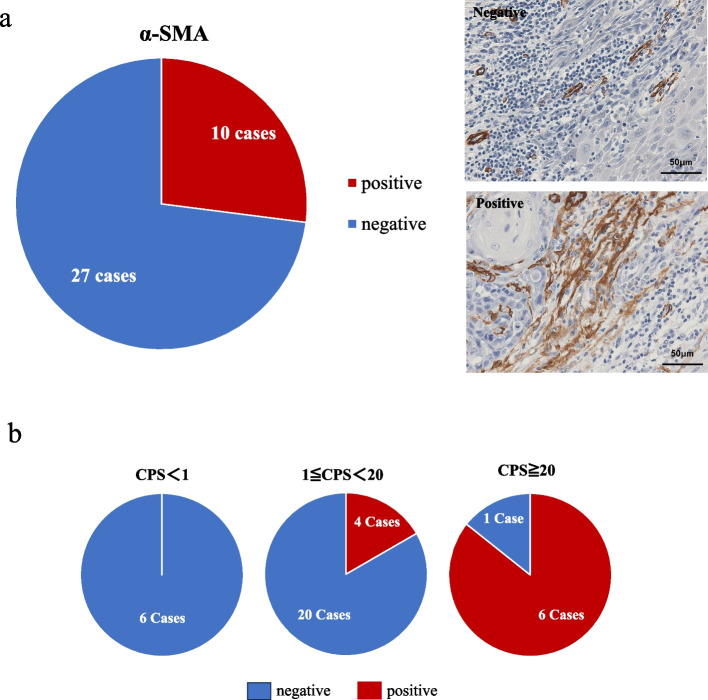
**a** Percentage of α-SMA positive in immunohistochemistry. Upper: Representative case of α-SMA negative, magnificationX400, scale bar 50 μm(without endothelial vascular cell). Lower: Representative case of α-SMA positive, magnificationX400, scale bar 50 μm. **b** Percentage of α-SMA positive among CPS arms

Evaluation of α-SMA, a marker of carcinoma-associated fibroblasts (CAFs), which are myofibroblasts constituting the cancer stroma with tumor-promoting potential, was positive in 10 cases (27%) (Fig. 3a). The positivity rates were 85.7% (6/7), 16.7% (4/24), and 0% (0/6), respectively, revealing a significant difference (*p* < 0.01) among the three groups (Fig. 3b).

## Discussion

The present study found a correlation between PD-L1 expression with p53 and a-SMA expression. Furthermore, both p53-positive and a-SMA-positive rates were identified to increase with higher CPS.

Higher PD-L1 positivity of lymphocytes in oral squamous cell carcinoma has been associated with poorer prognosis [11]. While the PD-L1 positivity rate was higher in T2 cases compared to T1 cases in our study, T3 and higher cases were not examined. The checkmate-048 study also reported an improvement in 1-year survival in patients with CPS ≥ 20 compared to cetuximab therapy [8]. However, in the present study, two patients with CPS ≥ 20 revealed recurrence, and subsequent treatment at other hospitals makes it certain whether immune checkpoint inhibitors were utilized.

p53 has been shown to play roles in apoptosis, DNA repair, and cell cycle regulation in cancer cells [12]. p53 mutations are found in most cancer types, including oral squamous cell carcinoma, and have been reported to be a poor prognostic factor [13]. Tojyo et al. previously reported a correlation between PD-L1 expression and mutant p53 expression in oral squamous cell carcinoma [14]. Additionally, PD-L1 is overexpressed in patients with malignant transformation of oral leukoplakia [15], which is possibly related to p53 mutations. However, it remains unclear whether wild-type p53 regulates PD-L1 expression, and it has been reported that miR-34a suppresses PD-L1 expression in breast cancer [16]. With these, Tojyo et al. predicted that wild-type p53 suppresses PD-L1 expression; however, mutation abolishes this function and upregulates PD-L1 expression, leading to a correlation between p53 and PD-L1 expression [14].

CAFs, a main component of tumor stroma, are shown to be poor prognostic factors in oral cancer [17–19]. These are considered to have various subtypes; however, one of the representative markers is α-SMA, a myofibroblast marker, and α-SMA positive CAFs have been reported to have various tumor-promoting properties [20]. CAFs have also been reported to be observed in esophageal squamous epithelial neoplasia and CIS [21]. Furthermore, growth factors derived from CAFs promote proliferative potential during the carcinogenic process [22]. In this study, only one case of eleven CIS cases showed the presence of α-SMA-positive fibroblasts. CAFs Although PD-L1 expression is induced by CAFs in lung adenocarcinoma [23], no reports on PD-L1 expression in oral cancer have been conducted to the extent of our research efforts. C-X-C motif chemokine ligand (CXCL) [2, 5] has been reported to be involved in the mechanism by which CAFs regulate PD-L1 expression [23, 24]. Meanwhile, PD-L1 has been reported to transform lung fibroblasts into myofibroblasts [25]. These aforementioned findings suggest that CAFs and PD-L1 may regulate each other's expression via cell–cell interactions. The ratio of CD8-positive T cells to CAFs may be useful in predicting prognosis and immunotherapy response [26], and further exploration of the relationship between CAFs and PD-L1 expression in oral cancer is necessitated. In contrast, among α-SMA-positive CAFs, the existence of CAFs involved in immunotherapy resistance is becoming clear, but with still many unknowns [20].

As mentioned previously, a limitation of this study included not examining patients with advanced disease beyond T3, and stage III and IV patients exhibited higher PD-L1 expression than stage I and II patients [11]. Only one case of cervical lymph node metastasis out of 37 cases was considered in this study, which poses challenges in capturing the characteristics of the cases. Additionally, since no cases were identified to have actually undergone immune checkpoint therapy, reflecting the actual treatment effect was not feasible. Hence, further investigation is recommended for staining patterns in patients with recurrent disease, cervical lymph node metastases, and postoperative immunotherapy, and increase the number of variations of cases to provide more robust evidence for our findings.

## Conclusion

The study findings suggest that PD-L1 expression is upregulated by cell–cell interaction in p53-positive or α-SMA-positive oral squamous cell carcinoma cases.

## Data Availability

The datasets during and/or analyzed during the current study available from the corresponding author on reasonable request.

## Abbreviations

PD-1: Programmed cell death protein 1
PD-L1: Programmed cell death ligand 1
CPS: Combined positive score
SMA: Smooth muscle actin
IHC: Immunohistochemistry

## References

[1] Hinshaw DC, Shevde LA (2019) The tumor microenvironment innately modulates cancer progression. Cancer Res 79(18):4557–4566. 10.1158/0008-5472.CAN-18-3962 31350295 10.1158/0008-5472.CAN-18-3962 PMC6744958

[2] Mezawa Y, Orimo A (2016) The roles of tumor- and metastasis-promoting carcinoma-associated fibroblasts in human carcinomas. Cell Tissue Res 365(3):675–689. 10.1007/s00441-016-2471-1 27506216 10.1007/s00441-016-2471-1

[3] Hill BS, Sarnella A, D’Ano G, Zannetti A (2020) Recruitment of stromal cells into tumour microenvironment promote the metastatic spread of breast cancer. Semin Cancer Biol 60:202–213. 10.1016/j.semcancer.2019.07.028 31377307 10.1016/j.semcancer.2019.07.028

[4] Tang Q, Zhao S, Zhou N, He J, Zu L, Liu T et al (2023) PD-1/PD-L1 immune checkpoint inhibitors in neoadjuvant therapy for solid tumors (Riew). Int J Oncol 62(4):49. 10.3892/ijo.2023.5497 36866750 10.3892/ijo.2023.5497 PMC10019757

[5] Sharma P, Allison JP (2015) The future of immune checkpoint therapy. Science 348(6230):56–61. 10.1126/science.aaa8172 25838373 10.1126/science.aaa8172

[6] Ochi N, Yamane H, Takigawa N (2022) Predictive factors for the efficacy of immune checkpoint inhibitors against lung cancer. JJLC 62:355–362 10.2482/haigan.62.355

[7] De Marchi P, Leal LF, Duval da Silva V, Albino da Silva EC, Cordeiro de Lima VC, Reis RM (2021) PD-L1 expression by Tumor Proportion Score (TPS) and Combined Positive Score (CPS) are similar in non-small cell lung cancer (NSCLC). J Clin Pathol 74:735–740. 10.1136/jclinpath-2020-206832. 10.1136/jclinpath-2020-206832 33589532

[8] Burtness B, Harrington KJ, Greil R, Soulières D, Tahara M, de Castro G Jr et al (2019) Pembrolizumab alone or with chemotherapy versus cetuximab with chemotherapy for recurrent or metastatic squamous cell carcinoma of the head and neck (KEYNOTE-048): a randomised, open-label, phase 3 study. Lancet 394(10212):1915–1928. 10.1016/S0140-6736(19)32591-7 31679945 10.1016/S0140-6736(19)32591-7

[9] Mezawa Y, Daigo Y, Takano A, Miyagi Y, Yokose T, Yamashita T et al (2019) CD26 expression is attenuated by TGF-β and SDF-1 autocrine signaling on stromal myofibroblasts in human breast cancers. Cancer Med 8(8):3936–3948. 10.1002/cam4.2249 31140748 10.1002/cam4.2249 PMC6639198

[10] Yu X, Zhang X, Wang F, Lin Y, Wang W, Chen Y et al (2018) Correlation and prognostic significance of PD-L1 and P53 expression in resected primary pulmonary lymphoepithelioma-like carcinoma. J Thorac Dis 10(3):1891–1902. 10.21037/jtd.2018.03.14 29707344 10.21037/jtd.2018.03.14 PMC5906333

[11] Adamski ŁJ, Starzyńska A, Adamska P, Kunc M, Sakowicz-Burkiewicz M, Marvaso G et al (2021) High PD-L1 expression on tumor cells indicates worse overall survival in advanced oral squamous cell carcinomas of the tongue and the floor of the mouth but not in other oral compartments. Biomedicines 9(9):1132. 10.3390/biomedicines9091132 34572318 10.3390/biomedicines9091132 PMC8471659

[12] Pfeifer GP, Holmquist GP (1997) Mutagenesis in the P53 gene. BBA 1333(1):M1–M8. 10.1016/s0304-419x(97)00004-8 9294014 10.1016/s0304-419x(97)00004-8

[13] Liu R, Sun K, Wang Y, Jiang Y, Kang J, Ma H (2021) The effects of proliferating cell nuclear antigen and p53 in patients with oral squamous cell carcinoma: a systematic review and meta-analysis. Ann Transl Med 9(23):1739. 10.21037/atm-21-6133 35071433 10.21037/atm-21-6133 PMC8743711

[14] Tojyo I, Shintani Y, Nakanishi T, Okamoto K, Hiraishi Y, Fujita S et al (2019) PD-L1 expression correlated with p53 expression in oral squamous cell carcinoma. Maxillofac Plast Reconstr Surg 41(1):56. 10.1186/s40902-019-0239-8 31857991 10.1186/s40902-019-0239-8 PMC6892985

[15] Ries J, Agaimy A, Wehrhan F, Baran C, Bolze S, Danzer E et al (2021) Importance of the PD-1/PD-L1 axis for malignant transformation and risk assessment of oral leukoplakia. Biomedicines 9(2):194. 10.3390/biomedicines9020194 33669300 10.3390/biomedicines9020194 PMC7920045

[16] Deng S, Wang M, Wang C, Zeng Y, Qin X, Tan Y et al (2023) p53 downregulates PD-L1 expression via miR-34a to inhibit the growth of triple-negative breast cancer cell: a potential clinical immunotherapeutic target. Mol Biol Rep 50(1):577–587. 10.1007/s11033-022-08047-z 36352176 10.1007/s11033-022-08047-z

[17] Li YY, Tao YW, Gao S, Li P, Zheng JM, Zhang SE et al (2018) Cancer-associated fibroblasts contribute to oral cancer cells proliferation and metastasis via exosome-mediated paracrine miR-34a-5p. EBioMedicine 36:209–220. 10.1016/j.ebiom.2018.09.006 30243489 10.1016/j.ebiom.2018.09.006 PMC6197737

[18] Wang Y, Jing Y, Ding L, Zhang X, Song Y, Chen S et al (2019) Epiregulin reprograms cancer-associated fibroblasts and facilitates oral squamous cell carcinoma invasion via JAK2-STAT3 pathway. J Exp Clin Cancer Res 38(1):274. 10.1186/s13046-019-1277-x 31234944 10.1186/s13046-019-1277-x PMC6591968

[19] Ding L, Ren J, Zhang D, Li Y, Huang X, Hu Q et al (2018) A novel stromal lncRNA signature reprograms fibroblasts to promote the growth of oral squamous cell carcinoma via LncRNA-CAF/interleukin-33. Carcinogenesis 39(3):397–406. 10.1093/carcin/bgy006 29346528 10.1093/carcin/bgy006

[20] Mezawa Y, Orimo A (2022) Phenotypic heterogeneity, stability and plasticity in tumor-promoting carcinoma-associated fibroblasts. FEBS J 289(9):2429–2447. 10.1111/febs.15851 33786982 10.1111/febs.15851

[21] Zhibin X, Shijie W, Mingli W, Weiwei Z, Xiaoling W, Zhiming D (2013) TGFβ1 and HGF protein secretion by esophageal squamous epithelial cells and stromal fibroblasts in oesophageal carcinogenesis. Oncol Lett 6(2):401–406. 10.3892/ol.2013.1409 24137336 10.3892/ol.2013.1409 PMC3789106

[22] Neli AB, Eric GN, Harold M (2004) Stromal fibroblasts in cancer initiation and progression. Nature 432(7015):332–337. 10.1038/nature03096 15549095 10.1038/nature03096 PMC3050735

[23] Inoue C, Miki Y, Saito R, Hata S, Abe J, Sato I et al (2019) PD-L1 Induction by cancer-associated fibroblast-derived factors in lung adenocarcinoma cells. Cancers (Basel) 11(9):1257. 10.3390/cancers11091257 31462002 10.3390/cancers11091257 PMC6770125

[24] Li Z, Zhou J, Zhang J, Li S, Wang H, Du J (2019) Cancer-associated fibroblasts promote PD-L1 expression in mice cancer cells via secreting CXCL5. Int J Cancer 145(7):1946–1957. 10.1002/ijc.32278 30873585 10.1002/ijc.32278 PMC6767568

[25] Guo X, Sunil C, Adeyanju O, Parker A, Huang S, Ikebe M et al (2022) PD-L1 mediates lung fibroblast to myofibroblast transition through Smad3 and β-catenin signaling pathways. Sci Rep 12(1):3053. 10.1038/s41598-022-07044-3 35197539 10.1038/s41598-022-07044-3 PMC8866514

[26] Zheng X, Jiang K, Xiao W, Zeng D, Peng W, Bai J et al (2022) CD8+T cell/cancer-associated fibroblast ratio stratifies prognostic and predictive responses to immunotherapy across multiple cancer types. Front Immunol 13:974265. 10.3389/fimmu.2022.974265 36439099 10.3389/fimmu.2022.974265 PMC9682254

